# Global longitudinal strain in pre-symptomatic patients with mutation for transthyretin amyloidosis

**DOI:** 10.1186/s13023-024-03473-7

**Published:** 2024-12-05

**Authors:** Grazia Canciello, Stefano Tozza, Gaetano Todde, Maria Nolano, Felice Borrelli, Giovanni Palumbo, Raffaella Lombardi, Emanuele Cassano, Wanda Acampa, Giovanni Esposito, Fiore Manganelli, Maria Angela Losi

**Affiliations:** 1 Department of Advanced Biomedical Sciences, University Federico II of Naples, Naples, Italy; 2 Department of Neuroscience and Reproductive and Odontostomatological Sciences, University Federico II of Naples, Via S Pansini 5, 80131 Naples, Italy

**Keywords:** Global longitudinal strain, Hereditary transthyretin amyloidosis, Pre-clinical disease, Thermal quantitative sensory testing

## Abstract

**Background:**

Hereditary transthyretin (ATTRv) amyloidosis is rare, autosomal dominant disease with a fatal outcome if left untreated. Early stages detection is crucial for intervention. We aimed identifying early indexes of cardiac involvement and their eventual correlation with neurological indexes, in pre-symptomatic subjects with TTR gene mutation.

**Methods:**

Sixteen TTR-mutation carriers (mean age 51 ± 9 years, 6 males, 7 with Val30Met and 9 with Phe64Leu mutation) without left ventricular hypertrophy were studied. Predicted Age of Disease Onset (PADO) and time to PADO (Time-to PADO = PADO-age at evaluation) were computed. Subjects underwent: cardiological and echocardiographic assessment including global longitudinal strain (GLS); tactile and thermal quantitative sensory testing (QST); Perugini score by bone scintigraphy.

**Results:**

Time to PADO was 30 ± 15 years. Nine subjects showed abnormal GLS (> −20%), unrelated to age, LVMi, MWT, E/e’, NT-proBNP or Time-to PADO. QST findings were abnormal in most subjects. At a worse cold pain threshold corresponded a worse GLS (r = 0.786, *p* < 0.001). Perugini score was positive in 1 subject.

**Conclusions:**

GLS and QST findings support an early involvement of heart and small nerve fibers even many years before PADO. Interestingly, cardiac impairment seems to parallel that of small, nerve fibers, at least in the earliest stage of disease.

## Introduction

Hereditary Transthyretin amyloidosis (ATTRv, where "v" stands for "variant") is a rare condition caused by mutations in the transthyretin (TTR) gene. It is an autosomal dominantly inherited, debilitating, progressive, and potentially fatal multisystem disorder if left untreated [1]. ATTRv is primarily characterized by the deposition of amyloid fibrils, predominantly in the peripheral nerves and heart [2–4]. This leads to symptoms such as polyneuropathy, hypertrophic cardiomyopathy, or a combination of both. Additionally, patients may experience other manifestations, including gastrointestinal and kidney impairment, as well as ocular dysfunction [1], underlying as a multidisciplinary approach is essential for the management of ATTRv patients [5].

It is important to consider all relatives of individuals with ATTRv as potential carriers of the familial mutation [6]. If willing to undergo genetic testing and testing positive for the mutation, these individuals should be directed to neurological and cardiological follow-up for a comprehensive assessment and management [7].

Ongoing efforts are dedicated to identifying new early biomarkers that can indicate the progression from an asymptomatic state to the manifestation of the first signs of the disease [8]. This research aims to improve the timely detection and management of ATTRv, ultimately contributing to better patient outcomes.

For the early identification of disease progression in mutation carriers, quantitative sensory testing (QST) seems to be a useful method for documenting small fiber dysfunction before any objective electrophysiological signs of peripheral nervous system (PNS) involvement [9, 10]. However, in the setting of cardiac involvement, there is no data exploring cardiac function before hypertrophy develops. In other cardiac conditions, at the preclinical stage, strain, which refers to the amount of deformation or change in size of the myocardial (heart muscle) tissue during the cardiac cycle, has been capable of identifying areas of abnormal tissue function before overt cardiac disease[11].

Thus, we undertook this study to understand the role of myocardial strain in pre-symptomatic ATTRv carriers and eventually establish a relationship between strain and QST.

## Methods

### Population

We prospectively evaluated 21 subjects (age 52 ± 15 years, 41% women) carrying a pathogenic variant in TTR gene without any symptom and sign of ATTRv and with normal nerve conduction study (NCS).

### Definitions

Diabetes was defined in presence of a fasting plasma glucose > 125 mg/dl or anti-diabetic treatment. Obesity was defined as a BMI ≥ 30 kg/m2. Hypertension was defined in presence of a systolic or diastolic blood pressure ≥ 140 and ≥ 90 mmHg, respectively or antihypertensive treatment [12]. New York Heart Association (NYHA) functional class was classified in Class I: No dyspnoea; Class II: dyspnoea with moderate exertion; Class III: dyspnoea with minimal exertion, but no symptoms at rest; Class IV: dyspnoea at rest [13].

### Cardiological assessment

The subjects underwent a comprehensive cardiological assessment, encompassing anamnesis, clinical examination, evaluation of potential symptoms, and electrocardiography (EKG). 2D and Doppler echocardiography were conducted using Philips IE33 systems, and the resulting images were digitally stored for offline analysis by an experienced sonographer (GC). Standard cardiac dimensions were determined as the mean of three cardiac cycles.

Echocardiography procedures were consistent with previous descriptions applied in patients with hypertrophic cardiomyopathy [14, 15]. Specifically, the end-diastolic left ventricular (LV) thickness of the anterior and posterior septum and of lateral and posterior wall was measured from 2-dimensional parasternal long-axis images, taken at the level of the mitral valve leaflet tips, papillary muscle level and at apex. A maximal wall thickness (MWT) of 12 or more was interpreted as indicative of initial overt cardiomyopathy [16].

Left ventricular ejection fraction (EF) and left atrial volume (LAV) were calculated using biplane modified Simpson’s rule. Mitral inflow was analysed for peak E-wave using Pulse-Doppler, allowing for a spectral display of mitral annulus velocities at septal and lateral corners. E’ velocity was measured, and the E/E’ ratio was computed at both corners of the mitral annulus. Careful adjustments of gains and filters were made to eliminate background noise and ensure a clear tissue signal.

Global longitudinal strain (GLS) was determined from apical images, with frame rates maximized (ranging from 50 to 70 frames per second) by narrowing the sector to isolate individual walls. Offline image analysis employed commercial software (TomTec software) utilizing speckle tracking methodology, which tracks the movement of natural acoustic speckles in the myocardium from 2D grey-scale images. The endocardium was manually traced, and myocardial motion was tracked with automated software. Tracking quality was verified manually and using the software’s automated quality grading scale. Segments were rejected if adequate quality could not be obtained, despite manual correction.

In all subjects Perugini score was assessed by 99mTc-labeled bisphosphonate (HMDP) and pathological Perugini score was considered when equal to 2 or 3.

### Neurological assessment

QST was performed according to standardized protocol to study the function of C (warm stimuli), Aδ (cold stimuli) and Aβ (tactile stimuli) fibres, previously described [9]. Briefly, QST was performed in foot dorsum on the non-dominant side. The thermal QST was conducted through Thermal Sensory Analyzer II (TSA-2001, Medoc Ltd., Ramat Yishai, Israel) with the method of limits Cold detection threshold (CDT), warm detection threshold (WDT), cold pain (CP) and heat pain (HP) were assessed.

On the other hand, tactile QST was performed by using a standardized set of calibrated monofilaments (Aesthesio Precision Tactile Sensory Evaluator, DanMic Global LLC, San Jose, CA 95124, USA) Moving stepwise from the thicker towards the thinner filament, tactile threshold (TT) was defined as the thinnest filament perceivable at least 5 times out of 10. Age-matched Z-score [= (single patient X-healthy control mean)/healthy control SD] was calculated for each QST findings and a Z-score greater/lower than ± 2 was considered as abnormal value.

We computed the Predicted Age of Disease Onset (PADO) for each subject and we calculated the time to PADO (Time-to PADO = PADO-age at evaluation).

At neurological evaluation, a blood sample was taken, and, among other findings, glomerular filtration rate (GFR) was estimated by EPI formula, and NT-proBNP was measured.

### Statistics

Data were analyzed using SPSS (version 25.0; IBM-SPSS, Armonk, NY). Continuous variables were described as mean ± standard deviation. Categorical variables were described as number (percentage). Unpaired T test was used when appropriate. The χ2 test were used to compare categorical variables, with the Monte Carlo simulation to obtain exact p-values. Correlations were performed by Pearson correlation. A value of *p* < 0.05 was significant.

## Results

Among 21 asymptomatic carriers, 2 subjects were excluded because of LV MWT ≥ 12 mm. In the remaining 19 subjects, three showed bad echocardiographic windows, not allowing strain measurements. Thus, the final population was of 16 subjects (age 48 ± 14 years, 40% women). Seven subjects carried Val30met whereas the remaining 9 Phe64Leu mutation.

Baseline clinical findings were reported in Table 1. There were 3 subjects with 1 cardiovascular (CV) risk factor, whereas in 3 there were 2 CV risk factors. There were no subjects with chronic kidney disease at III or less stage. In addition, NT-proBNP was normal in all subjects.

**Table 1:** Baseline characteristics of carriers

Carrier	Age (years)	Sex	Muta-tion	PADO (years)	Time to PADO (years)	Obesity	Diabetes	Hypertension	GFR-EPI (ml/m/1.73m^2^)	NT-pro BNP (pg/ml)	Perugi-ni Score
1	54	M	Val30Met	72	18	No	No	Yes	98	8	0
2	49	M	Phe64Leu	60	11	No	No	No	99	49	0
3	45	M	Phe64Leu	65	20	No	No	Yes	103	22	0
4	56	M	Phe64Leu	65	9	Yes	No	Yes	73	36	0
5	26	F	Val30Met	45	19	No	No	No	126	18	0
6	27	F	Val30Met	45	18	Yes	No	No	122	18	0
7	37	M	Phe64Leu	66	29	No	No	No	89	8	0
8	35	M	Phe64Leu	64	29	No	No	No	93	24	0
9	45	F	Phe64Leu	64	19	Yes	No	No	77	136	0
10	35	M	Val30Met	60	25	No	No	No	118	31	0
11	73	M	Val30Met	55	−18	No	Yes	No	79	60	3
12	43	F	Val30Met	70	27	No	No	No	98	77	0
13	60	F	Phe64Leu	64	4	No	No	No	80	87	0
14	44	M	Val30Met	60	14	No	No	No	96	22	0
15	68	F	Phe64Leu	65	−3	No	Yes	Yes	90	121	1
16	50	M	Phe64Leu	65	15	Yes	No	Yes	100	164	0

*GFR-EPI* Glomerular filtration rate calculated by EPI formula, *PADO* Predicted age of disease onset

Cardiological, EKG, and echocardiographic evaluation are reported in Table 2. All subjects reported no dyspnea or other symptoms. Although the presence of normal LV ejection fraction, normal MWT and LV mass, 9 subjects showed abnormal GLS (i.e. > −20%). GLS was not related to age (r = −0.196; *p* = 0.468), to LVMi, (r = 0.473, *p* = 0.075), to MWT (r = 0.340, *p* = 0.198), to E/e’ (r = 0.124, *p* = 0.673) and to NT-proBNP (r = −0.112, *p* = 0.681). GLS was not related to Time-to PADO (r = −0.058, *p* = 0.830), with abnormal GLS already abnormal even in subjects with time-to PADO > 10 years. In addition, subjects with CV risk factors, such as obesity, hypertension and diabetes, had not different GLS from those without CV risk factor (−19.6 ± 1.3% vs −18.5 ± 2.3%, respectively, *p* = 0.253). Table 3 reported QST findings. As shown in the table, in the majority of pre-symptomatic subjects there were abnormal findings.

**Table 2:** Cardiological assessment in the studied population

Carrier	NYHA functional class	EKG	MWT (mm)	LVMi (g/m^2^)	LVEF (%)	LAVI (ml/m^2^)	E/e’	GLS (%)
1	I	Normal	10	71	60	25	7	−18
2	I	Normal	9	74	62	23	4	−20
3	I	Normal	10	78	68	25	8	−19
4	I	Normal	9	69	60	18	8	−15
5	I	Normal	8	64	62	25	6	−20
6	I	Normal	8	85	63	22	5	−21
7	I	Normal	9	96	60	31	4	−19
8	I	Normal	8	72	55	23	7	−18
9	I	Normal	9	78	59	23	9	−18
10	I	Normal	8	69	60	31	12	−20
11	I	Normal	9	89	68	24	8	−16
12	I	Normal	8	59	63	27	6	−18
13	I	Normal	9	46	57	33	7	−22
14	I	Normal	8	77	61	19	8	−20
15	I	Normal	7	59	80	32	7	−22
16	I	Normal	11	71	64	26	8	−19

*EKG* Electrocardiogram, *GLS* Global longitudinal strain, *LAVI* Left atrial volume index, *LVMi* Left ventricular mass index, *LVEF* Left ventricular ejection fraction, *MWT* Maximal wall thickness, *NYHA* New York Heart Association

**Table 3:** Neurological assessment in the studied population

Carrier	Quantitative sensory testing at dorsal foot (°C)									
	Tactile Threshold	Abnormal Tactile Threshold	Cold Detection Threshold	Abnormal Cold Threshold	Warm Detection Threshold	Abnormal Warm Threshold	Cold Pain Threshold	Abnormal Cold Pain Threshold	Heat pain threshold	Abnormal heat pain threshold
1	0.40	no	4.50	no	11.20	yes	15.30	yes	29.40	yes
2	1.00	yes	9.80	yes	13.30	yes	17.60	yes	26.90	yes
3	0.60	no	3.40	no	3.40	yes	14.50	yes	28.60	yes
4	0.60	no	13.30	yes	11.30	yes	12.70	yes	32.00	yes
5	0.40	no	4.30	yes	4.20	no	8.10	no	6.30	no
6	0.40	no	1.70	no	2.80	no	4.90	no	4.50	no
7	1.00	yes	3.80	yes	12.50	yes	15.40	yes	17.00	yes
8	0.60	no	8.90	yes	8.30	yes	15.80	yes	31.60	yes
9	0.40	no	11.00	yes	13.10	yes	17.50	yes	32.00	yes
10	0.16	no	9.40	yes	12.60	yes	16.10	yes	18.80	yes
11	0.40	no	3.30	no	6.80	no	32.00	Yes	15.60	yes
12	4.00	yes	4.20	yes	3.20	no	7.60	no	29.50	yes
13	1.00	no	7.10	yes	4.30	no	5.70	no	7.90	no
14	0.07	no	0.56	no	4.20	yes	11.40	yes	2.10	no
15	1.40	yes	5.10	no	9.70	yes	17.20	yes	1.00	yes
16	1.00	no	18.70	yes	15.10	yes	17.50	yes	32.00	yes

**Fig. 1:**
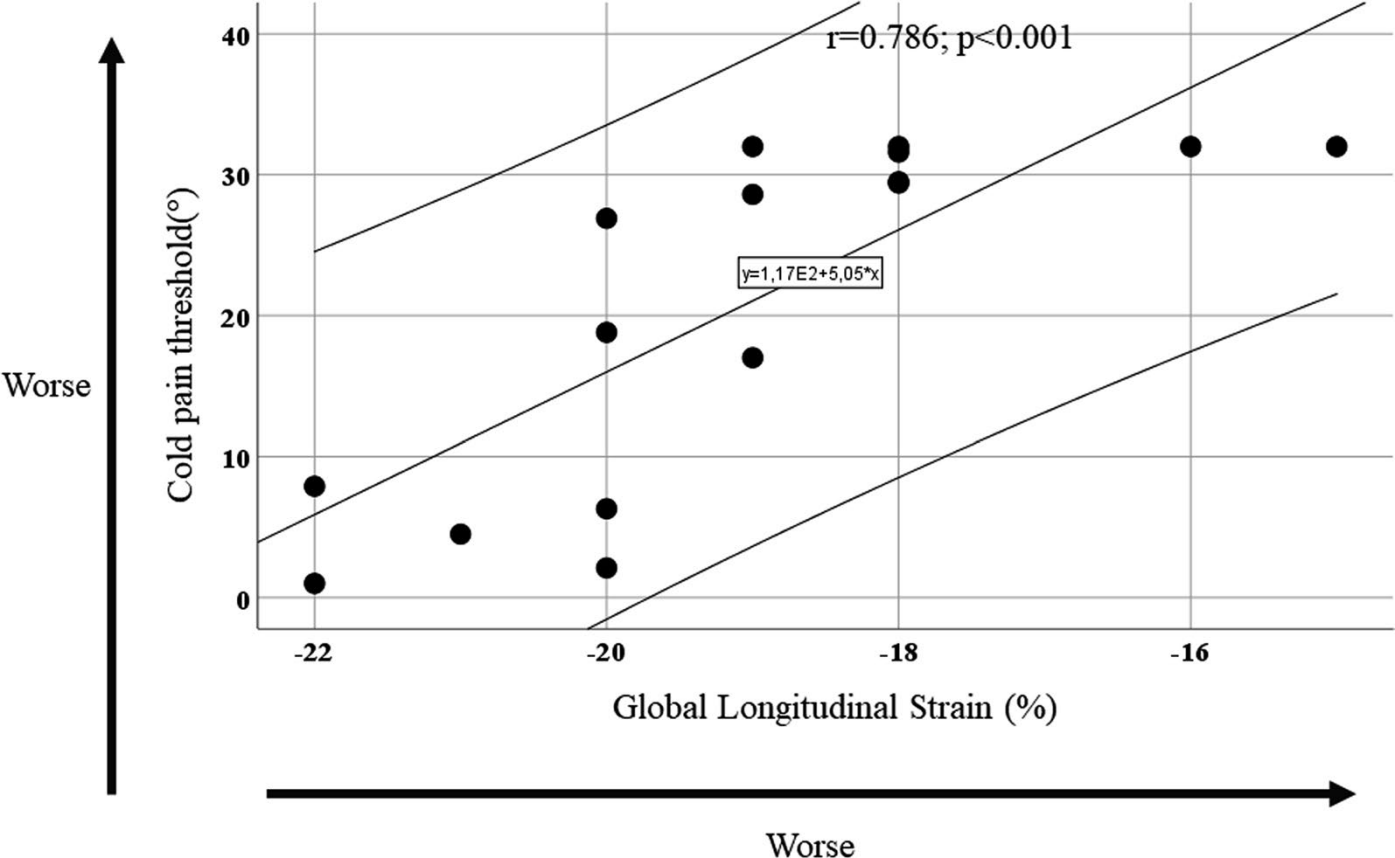
Scatterplot of global longitudinal strain and cold pain threshold. Higher values of longitudinal strain indicate worse systolic myocardial deformation, whereas higher values of cold pain threshold indicate a greater amount of Aδ fibre dysfunction in detection of cold pain

Cardiological and neurological correlation. Table 4, reports differences in GLS in normal and abnormal QST findings, showing that GLS was lower in subjects with abnormal QST findings, however reaching statistical significance only in those with abnormal cold pain threshold (Table 4). In addition, GLS was positively correlated to cold pain threshold, demonstrating that at worse cold pain threshold a worse deformation was present (r = 0.786, *p* < 0.001) (Fig. 1). Perugini score was 0 in 14 subjects, 1 in one, and 3 in the reaming subject. Among the 14 subjects with Perugini score of 0, GLS was abnormal in 8 of them, the subject with Perugini score 1 showed normal GLS, whereas, the remaining subject with Perugini score of 3, showed abnormal GLS. The correlation between cold threshold and GLS holds true even when we excluded the two patients with positive Perugini score, that were also the two patients with diabetes (r = 0.760, *p* = 0.002).

**Table 4:** Differences of GLS in normal and abnormal quantitative sensory testing

	Tactile threshold (°)			Cold threshold (°)			Warm threshold (°)			Cold pain threshold (°)			Heat pain threshold (*)		
	Normal (# 12)	Abnor-mal (#4)	*p*	Normal (#6)	Abnor-mal (#10)	*p*	Normal (#6)	Abnor-mal (#10)	*p*	Normal (#4)	Abnor-mal (#12)	*p*	Normal (#4)	Abnor-mal (#12)	*p*
GLS (%)	−18.8 ± 2.0	−19.8 ± 1.7	0.43	−19.3 ± 2.2	−18.9 ± 1.9	0.68	−19.3 ± 2.2	−18.9 ± 1.9	0.68	−20.8 ± 1.0	−18.5 ± 1.8	0.04	−20.3 ± 1.7	−18.7 ± 1.9	0.16

*GLS* Global longitudinal strain

The central figure reports the main methods and results of our paper.

## Discussion

To the best of our knowledge, this is the first study investigating myocardial strain imaging in absence of increased MWT, and the relationship between strain imaging and neurological findings in asymptomatic ATTR mutation carrier. We found that LV strain was reduced in more than 50% of the asymptomatic mutation carrier. By analysing by two-dimensional echocardiography overall LV segment, we were able to exclude subjects with MWT ≥ 12 in any of the LV segments, which is of particular importance in the light of the reported increased MWT in LV region other than interventricular septum [17]. Thus, in our population we can exclude that hypertrophy was the cause of reduced strain.

In pre-symptomatic stage, which refers to the early stages of a disease [18, 19], myocardial strain can offer several advantages such as subtle changes in myocardial function before traditional measures, such as ejection fraction, are abnormal [11]. This can be crucial in identifying cardiac dysfunction at an early, potentially reversible stage. The reduction of strain, in our opinion was due to the presence of mutation. Firstly, although it is generally recognized that myocardial strain tends to decrease with increasing age [20], because the heart undergoes various structural and functional changes over the course of a person's life, in our population with various age, there were no correlation between GLS and age. Secondly, the presence of CV risk factors did not affect GLS as well [21], again indicating that the mutation per se was responsible for GLS alterations. In addition, more than 50% of our population showed reduced GLS, i.e. > −20%. The normal value of GLS has been largely debated over time, because depending, first, on the vendor used for acquisition and measurement [22]. However, guidelines, even pointing out this limitation, indicate a value > −20% as pathological [22]. Thereafter, this value has been confirmed in a meta-analysis involving 24 studies with more than 2000 subjects studied by strain echocardiography, confirmed a value > −20% as pathological. The potential significance of this reduction is not demonstrable with the present analysis; however, it underscores the fact that myocardial properties are already pathological many years before PADO.

The presence of a Perugini score of 1 in one of our patients currently excludes the suspicion of ATTR cardiomyopathy. More intriguing is the presence of a Perugini score of 3 in another patient, despite the absence of increased LV MWT. According to current recommendations [6], this patient is not considered to have cardiac amyloidosis because hypertrophy is necessary to initiate further testing, such as bone scintigraphy.

Since the utility of bone scintigraphy as an indicator of cardiac involvement during the preclinical phase of the disease, i.e., in the absence of LV hypertrophy, is not well understood, we typically perform this test. While bone scintigraphy has proven indispensable for diagnosing ATTR cardiomyopathy in populations with a high pre-test likelihood, it remains unproven as a screening methodology [23].

Moreover, our findings suggest that cardiac and neural damage might go parallel in pre-symptomatic stage since a direct correlation was found between GLS and CP, which represents the sensory modality more frequent abnormal in ATTRv carriers [9]. The amyloidogenic cascade is an intricate process that remains incompletely understood, with various mechanisms at play [24]. In vitro, the conversion of TTR into amyloid fibrils initiates when the stable tetrameric form of TTR becomes destabilized, leading to the dissociation of the protein into dimers and monomers that adopt a non-native conformation [25]. Factors contributing to TTR instability and a shift towards the monomeric state include genetic mutations [25].

Misfolded monomers subsequently self-assemble into soluble, non-fibrillar oligomers, believed to be precursors to amyloid fibrils, exerting significant cytotoxic effects on tissues. Over time, these misfolded proteins aggregate and accumulate as amyloid deposits. This sequence of events culminates in the formation of a nucleus with sufficient stability to grow through the addition of monomers [26]. This stochastic process occurs during a specific phase known as the lag or nucleation phase [26]. The introduction of preformed seeds can notably expedite or even complete this phase, a phenomenon referred to as seeding [26, 27]. In the subsequent elongation phase, the addition of monomers to the nucleus results in the formation of amyloid fibrils [1]. The remarkably slow rate of progression, measured in years, observed in vitro suggests the involvement of catalysing factors in vivo [28]. Of course, we cannot demonstrate at what of these stages are our carriers, however, we can suppose that before neurological and/or cardiac symptoms are present as well as before any objective electrophysiological or echocardiographic signs of PNS or heart involvement are detectable, there are subclinical neurological alteration paralleling those of the heart.

In fact, this is the first study that has compared the cardiac and nervous system evaluation in the ATTRv pre-symptomatic group. In such rare and progressive disease, a multidisciplinary approach [5] should be guaranteed especially in the carrier group. In fact, a close inter-specialty collaboration is essential to determine the “converted” state of the patients and thus the optimal treatment choice.

In the last decade, great effort was done in evaluating several instrumental tests, both conventional (e.g. NCS, Sudoscan) or unconventional (QST, skin biopsy, serum biomarker as Nefl, nerve ultrasound, MRI neurography), useful to precociously detection of the nerve involvement [29]. In this study, two unconventional tests evaluating both cardiac and nerve involvement (GLS and QST), was performed to precociously detect multisystemic involvement. All the 9 patients with abnormal GLS (> −20) had at least one abnormal QST parameter as well.

With the development of disease-modifying therapies such as TTR stabilizers and gene silencing agents, early detection of ATTRv amyloidosis has become more critical than ever. These therapies offer the potential to alter the natural course of the disease significantly, particularly when initiated early. Therefore, increased awareness, improved diagnostic strategies, and proactive family screening are essential components in the management of ATTRv amyloidosis to maximize the therapeutic benefits and improve patient outcomes.

It is questionable if subject displaying abnormal QST findings, can be considered as affecting by small fibre neuropathy without symptoms and thus they have developed amyloidosis. However, the expert consensus considers a carrier “converted” when two instrumental tests resulted abnormal also in absence of any symptoms [30]. Can carriers, displaying abnormal QST and GLS, be considered as “converted”? Can they access to a ATTRv treatment (tetramer stabilizer or gene silencers)? Are they at risk of soon conversion? These questions are still unsolved and only the follow-up of these patients can clarify the usefulness of a combined approach with GLS and QST in the “conversion” diagnosis.

## Study limitations

One significant limitation of this study is attributed to the relatively small cohort size, a consequence of the rarity of the disease under investigation. While efforts were made to include as many eligible participants as possible, the inherent scarcity of cases constrained the overall sample size.

Furthermore, it's important to note that biopsy procedures were not conducted in the enrolled patients. This decision was based on the consensus that biopsy, while often a standard diagnostic tool in similar contexts, was deemed unnecessary or overly invasive in the current clinical scenario. Furthermore, half of our population is characterized by Phe64Leu carriers, in whom pathological amyloid deposits in nerve biopsies or abdominal fat needle aspiration are not always detectable [31].

Looking ahead, there is a possibility to incorporate MRI into future assessments.

MRI holds promise as a non-invasive imaging modality that could offer valuable insights into disease progression, phenotype characterization, and treatment response. Consideration of MRI in future research endeavours could enhance our understanding of the disease trajectory and aid in refining diagnostic and therapeutic strategies. However, it's imperative to acknowledge that this potential avenue for investigation remains speculative at this stage and would require careful planning and execution in subsequent studies.

## Conclusion

In conclusion, this is the first multidisciplinary study that evaluated cardiological and neurological features in ATTRv carriers. GLS and QST findings demonstrated an early involvement of heart and small nerve fibres even many years before PADO with a parallel impairment among the two systems (cardiac and small fibres) at least in the earliest stage of disease.

## Data Availability

Data are not publicly available due to their containing information that could compromise the privacy of research participants. De-identified data are available upon reasonable request to the corresponding author.

## Abbreviations

ATTRv: Hereditary transthyretin amyloidosis
CDT: Cold detection threshold
CP: Cold pain
CV: Cardiovascular
EF: Ejection fraction
EKG: Electrocardiography
GFR: Glomerular filtration rate
GLS: Global longitudinal strain
HMDP: 99MTc-labeled bisphosphonate
HP: Heat pain
LAV: Left atrial volume
LV: Left ventricular
MWT: Maximal wall thickness
NYHA: New York heart association
PADO: Predicted age of disease onset
PNS: Peripheral nervous system
QST: Quantitative sensory testing
TT: Tactile threshold
WDT: Warm detection threshold
